# The Virion Host Shut-Off (vhs) Protein Blocks a TLR-Independent Pathway of Herpes Simplex Virus Type 1 Recognition in Human and Mouse Dendritic Cells

**DOI:** 10.1371/journal.pone.0008684

**Published:** 2010-02-18

**Authors:** Christopher R. Cotter, Marie L. Nguyen, Jacob S. Yount, Carolina B. López, John A. Blaho, Thomas M. Moran

**Affiliations:** 1 Department of Microbiology and Immunology Institute, Mount Sinai School of Medicine, New York, New York, United States of America; 2 Department of Microbiology and Immunology, Des Moines University, Des Moines, Iowa, United States of America; New York University, United States of America

## Abstract

Molecular pathways underlying the activation of dendritic cells (DCs) in response to Herpes Simplex Virus type 1 (HSV-1) are poorly understood. Removal of the HSV virion host shut-off (vhs) protein relieves a block to DC activation observed during wild-type infection. In this study, we utilized a potent DC stimulatory HSV-1 recombinant virus lacking vhs as a tool to investigate the mechanisms involved in the activation of DCs by HSV-1. We report that the release of pro-inflammatory cytokines by conventional DC (cDC) during HSV-1 infection is triggered by both virus replication-dependent and replication-independent pathways. Interestingly, while vhs is capable of inhibiting the release of cytokines during infection of human and mouse cDCs, the secretion of cytokines by plasmacytoid DC (pDC) is not affected by vhs. These data prompted us to postulate that infection of cDCs by HSV triggers a TLR independent pathway for cDC activation that is susceptible to blockage by the vhs protein. Using cDCs isolated from mice deficient in both the TLR adaptor protein MyD88 and TLR3, we show that HSV-1 and the vhs-deleted virus can activate cDCs independently of TLR signaling. In addition, virion-associated vhs fails to block cDC activation in response to treatment with TLR agonists, but it efficiently blocked cDC activation triggered by the paramyxoviruses Sendai Virus (SeV) and Newcastle Disease Virus (NDV). This block to SeV- and NDV-induced activation of cDC resulted in elevated SeV and NDV viral gene expression indicating that infection with HSV-1 enhances the cell's susceptibility to other pathogens through the action of vhs. Our results demonstrate for the first time that a viral protein contained in the tegument of HSV-1 can block the induction of DC activation by TLR-independent pathways of viral recognition.

## Introduction

Herpes simplex virus (HSV) is a highly efficient pathogen that infects between 60 and 80% of the US population [1]. An important aspect of this pathogen's success is the ability to suppress or evade the host immune response at multiple stages of the infection. Early in a primary infection, HSV-infected epithelial cells are shielded from the host immune system long enough to produce progeny virions that infect nearby sensory neurons (reviewed in [2]). The virus enters a latent state in these neurons in which limited gene expression occurs. Although the host immune response eventually overcomes the viral immune suppressive activities and clears the HSV infected epithelial cells, it is unable to remove latent viral genomes from the neuronal tissues. The host immune response against HSV greatly impacts the severity of primary lesions [3], [4], [5] and is likely involved in the frequency of reactivation [6], [7].

Upon infection of the epithelial tissues, HSV encounters a specialized type of immune cell, the dendritic cell (DCs). These cells are the most potent antigen presenting cells known [8]. In an uninfected individual, DCs reside within the epithelial tissue and circulate in the blood stream in an immature state. Upon proper stimulation, they undergo a process referred to as maturation characterized by increased pro-inflammatory cytokine secretion and expression of certain surface markers, e.g., CD80 and CD86 [9]. Concurrent with this maturation event, the DCs migrate into lymph nodes where they can present antigen and stimulate T-cells. HSV has been shown to infect and replicate to low levels within immature [10] but not mature DCs [11], [12]. Moreover, HSV-infected DCs show a decrease in surface molecule expression and cytokine release upon stimulation leading to a reduced ability to stimulate allogeneic T-cell proliferation [10], [13], [14], [15]. The mechanism(s) underlying the suppression of DC activation by HSV have not been deciphered. Identification of the viral genes involved in suppressing DC maturation is an active area of research that has implications for vaccine design and anti-HSV immunotherapy.

Pathogen recognition by innate immune cells of the host is a critical first step for the generation of an effective immune response. The cellular pattern recognition receptors (PRRs) necessary for the response to HSV vary based on cell type and tissue localization. TLR9 signaling has been shown to be responsible for splenic pDC activation in response to HSV-1 infection [16]. Recognition of a virion component at the cell surface can activate signaling through TLR2 in microglial cells [17]. Additionally, TLR2 is important for cytokine release in certain cell systems and as such is a determinant for herpes encephalitis [18]. A role for RIG-I in cooperation with TLR9 in the recognition of HSV-2 has also been shown for fibroblast and macrophage cell lines [19]. Further, TLR3 is critical for protection against severe herpes infections and implicated as a target of viral anti-apoptotic genes during human monocytic cell infections [20], [21]. An additional level of complexity is added by the fact that a minor sub-population of virus within certain wild-type HSV-1 KOS viral stocks has been reported to utilize both TLR2 and TLR9 to activate cDCs [22].

The virion host shutoff (vhs) protein of HSV is an RNAse, which is known to preferentially degrade mRNA species [23], [24], [25], [26], [27]. It is encoded by the U_L_41 gene, and is synthesized late in infection (after genome replication) [28]. Vhs is also present in the tegument and thus immediately functional following virion uncoating [23], [24], [25]. The nuclease function of the *de novo* synthesized vhs is thought to be partially compromised as a result of its association with another viral protein, VP16 [29], [30]. One earlier study showed that vhs blocks DC maturation. In this report, the authors found that DCs non-productively infected with a virus simultaneously lacking the ICP4, ICP27, γ34.5, VP16, and vhs genes displayed higher levels of cytokine secretion and T-cell stimulation ability than a similar virus mutant containing wild type vhs [31].

Here we directly determined the role of the vhs protein present in the virus tegument on DC maturation in the context of a productive HSV-1 infection using a recombinant HSV-1 virus deficient in only vhs. In addition, the vhs deficient virus was used as a tool to identify molecular pathways involved in the triggering of DC maturation by HSV. Our data suggests that vhs functions primarily to prevent DC activation by a TLR independent pathway of virus recognition.

## Materials and Methods

### Viruses and TLR Agonists

**Figure 1:**
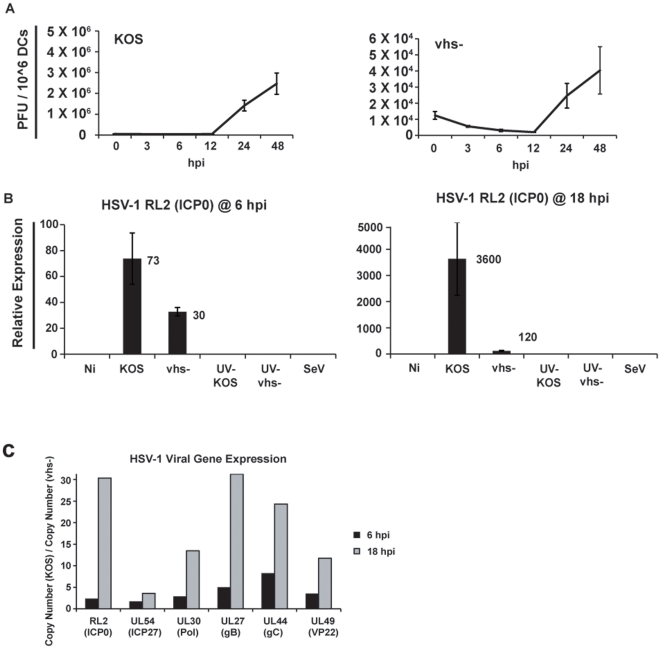
HSV-1 productively infects and replicates in human cDCs. (a.) Human monocyte-derived conventional dendritic cells (hu-cDCs) were infected with wild type HSV-1[KOS 1.1] (KOS) or the vhs-deficient virus, vhs-Δsma (vhs-), at an MOI of 5. Media of infected cultures was collected at various times post infection and assayed for infectious virions using a vero cell plaque assay. The mean and standard deviation of triplicate assays are plotted. (b.) qRT-PCR looking at mRNA levels of the immediate early viral gene RL2 at 6 hpi and 18 hpi. For UV-treatments, virus stocks were exposed to UV light at a distance of 10 cm for 4 minutes. Both live and UV-treated viruses were used to infect cDCs at an MOI of 5. SeV-C infection was performed at an MOI of 1.5. Data is represented as relative expression to normalized housekeeping gene levels. (c.) The ratio of the relative copy numbers for a series of HSV-1 viral genes (KOS/vhs-) was determined for hu-cDCs at 6 hpi and 18 hpi.

KOS 1.1, referred to in this study as KOS, was the wild-type strain of HSV-1 used in all experiments. The vhs mutant, Δsma, referred to in this study as vhs^−^, was generously provided by G. Sullivan Read and has been previously described [27]. It contains a deletion of the 588 basepair region between SmaI restriction enzyme sites in the U_L_41 gene. HSV-GFP was generously provided by Dr. D. Leib and contains the insertion of GFP between UL49 and UL50 of the parental KOS strain. All viruses were propagated and quantified on Vero cells as previously described [32], [33]. For experiments requiring UV inactivated viruses, viral stocks were exposed to UV irradiation at 10 cm from a germicidal lamp (UVP Multiple-Ray 8-WUV lamp [60 Hz]; Fisher) for 4 minutes. This amount of UV irradiation has previously been shown to prevent viral transcription and replication without inactivating the function of a representative tegument protein, VP16 [34]. To confirm UV inactivation in the present study, transcript levels for viral immediate early gene products and RL2 (ICP0) were shown to be absent compared to non-UV inactivated stocks via qRT-PCR (Fig. 1b). Further, no virus growth was observed during Vero cell plaque assay. Recombinant Newcastle Disease virus (NDV) B1 was generated from B1 Hitchner avian vaccine strain as previously described [35]. Sendai Virus (Cantel strain) was grown in 10-day old embryonated eggs as previously described [36]. pI:C was obtained from Amersham Biosciences and utilized at a concentration of 250 µg/mL. CpG was obtained from Coley Pharmaceutical Group and utilizied at a concentration of 6 µg/mL. LPS was obtained from Alexis Biochemicals and utilized at a concentration of 300 ng/mL.

### Mice

Wild-type C57/B6 mice were obtained from Taconic Farms. MyD88−/− were kindly provided by Dr. S. Akira (Osaka University, Osaka, Japan) and Dr. R. Steinman (Rockefeller University, New York, NY). TLR3−/− mice were obtained from Dr. R. Flavell (Yale University, New Heaven, CT). MyD88/TLR3 double knockout mice were obtained by crossing single knockouts until homozygous for both genes. All mice were bred and maintained in our facility consistent with regulations of animal care standard protocol as described by the Mount Sinai School of Medicine IACUC committee.

### Cells

#### Vero cells

Vero cells were grown in tissue culture medium (Dulbecco's modified Eagles medium [Mediatech]) with 10% fetal calf serum [Hyclone], 50 µg/ml gentamycin [Invitrogen]. All cells were grown at 37°C at 5–7% CO2.

***Human monocyte-derived conventional dendritic cells (hu-cDCs)*** were cultured from peripheral blood monocytes as previously described [37]. Briefly, cells were isolated by Ficoll density gradient separation from Buffy coats (30–60 ml each) of healthy human donors (Mount Sinai Blood Center and New York Blood Center). CD14+ monocytes were isolated using magnetic bead separation with anti-CD14 antibody-labeled magnetic beads and iron-based MidimacsLS colums (MiltenyiBiotec). Cells were cultured for 5–6 days in RPMI containing 10% fetal bovine serum (FBS), 100 U/ml penicillin, 100 µg/ml streptomycin, 500 U/l human granulocyte-macrophage colony-stimulating factor (GMCSF), and 1,000 U/ml human interleukin-4 at 10^6^ per ml prior to infection.

***Human plasmacytoid dendritic cells (hu-pDCs)*** were directly isolated from the peripheral blood mononuclear cell (PBMC) fraction after Ficoll density separation of Buffy coats using BDCA4 (CD304)+ antibody labeled magnetic beads and elution through iron-based MidimacsLS columns (MIltenyi Biotec). Sample purity was assessed using Flow Cytometry. Briefly, BDCA4+ cells were stained with FITC-CD123 and PE-BDCA2 (CD303), as per manufacturer's instructions. Marker expression was measured using the FC500 flow cytometer from Beckman Coulter; data analysis was conducted using Flowjo software. Purity was greater than 95%.

***Human naïve CD4+ T-cells*** were directly isolated via negative selection from the PBMC using an antibody cocktail (Miltenyi Biotec) that contained biotin-conjugated CD8, CD14, CD16, CD19, CD36, CD45RO, CD56, CD123, TCRγ/δ, and Glycophorin A. Cells were further purified to remove HLA-DR+ cells using anti-HLA-DR microbeads (Miltenyi Biotec). Isolations were conducted using iron-based MidimacsLS columns (MIltenyi Biotec).

***Human CD8+ T-cells*** were isolated similar to CD4+ T-cells by antibody depletion of all non-CD8+ cells followed by elution through iron-based MidimacsLS columns (MIltenyi Biotec).

***Murine-derived dendritic cells (mu-cDCs)*** were cultured from bone marrow derived monocytes as previously reported [38]. Briefly, bone marrow was extracted from mouse femurs and tibias. Red blood cells were lysed with ammonium chloride buffer. MHC class II-expressing cells and lymphocytes were removed using a cocktail of antibodies and magnetic bead separation. Purified monocytes were cultured in 24 well dishes in RPMI medium containing 10% FBS, 50 µg/ml gentamycin, 2 mM glutamine, 1 mM Na Py, and 50 U/ml recombinant granulocyte macrophage-colony-stimulating factor (PreproTech EC) at 1×10^6^ cells/ml.

***Murine-derived plasmacytoid dendritic cells (mu-pDCs)*** were prepared by Flt3-treatment of mouse bone marrow following RBC lysis. Flt3 was added at a concentration of 100 ng/mL. Cells were cultured for 10 days at 37°C; media was replaced containing Flt3 on day 5. Cells were sorted and enriched for CD11b-, B220+ cells on day 10.

### Multiplex ELISA

Cells and debris were removed from media of infected cells by centrifugation (1000 x g, 5 minutes). Between 25 and 50 µl of this cleared supernatant was subjected to multiplex ELISA procedure for human IL-2, human IL-4, human IL-5, human IP-10 as per manufacturer's (Upstate) instructions. Briefly, the supernatants were mixed with a combination of antibody-coated beads with specific fluorescence properties. After appropriate washing, the beads were mixed with PE-conjugated antibodies that will bind to the cytokine of interest. A final set of washes was completed and the fluorescent properties of each sample and appropriate standards were measured using a Luminex plate reader. The data was analyzed using software from Applied Cytometry Systems.

### Capture ELISAs

ELISA kits for human TNF-α and human and mouse IL-6 (DuoSet ELISA development systems) were obtained from R&D Systems. ELISA kits for human IL-12p70 and human IFN-γ were obtained from eBiosciences. ELISAs for mouse IL-12p40 were conducted using capture and secondary antibodies from BD Pharmingen. Manufacturer instructions were followed for all ELISA methods used. Plates were read on an ELISA reader from Bioteck Instruments.

### Flow Cytometry and Antibodies

Mouse monoclonal antibodies against both HSV-1 VP5 and β-actin were obtained from Santa Cruz. PE-conjugated CD86 and FITC-conjugated CD80 were obtained from Beckman Coulter and BD Pharminigen, respectively. Cells were stained with antibodies and subject to flow cytometry using the FC500 flow cytometer from Beckman Coulter Data was analyzed with Flowjo software.

### Quantitative RT-PCR (qRT-PCR)

RNA isolation was conducted on infected and non-infected cell cultures at the indicated time-points using the commercially available Absolutely RNA Microprep Kit from Stratagene. RNA was concentrated via precipitation with ammonium acetate. An equal amount of RNA was reverse transcribed to generate the cDNA template to be used in qRT-PCR reactions using oligodT (Roche) and Affinity Script Reverse Transcriptase (Stratagene). cDNA was diluted 50-fold in water. PCR reactions were conducted in triplicate using specific primers, Platinum Taq Polymerase (Roche), and SYBR green (Roche). Primer sequences are shown in Table 1. Reactions were run on 384 well plates using the Roche Lightcycler; normalizations were conducted using the levels of the housekeeping genes β-Actin, α-Tubulin, and rps11.

**Table 1:** Primer Sequences.

Gene	Sense Sequence (5′ →3′)	Anti-sense Sequence (5′ →3′)
HSV-1 RL2 (ICP0)	GTGGTCGCCCTGTCGCGTTA	GTCGCCATGTTTCCCGTCTG
HSV-1 UL54 (ICP27)	CCCTTTCTCCAGTGCTACCT	CTCTCCGACCCCGACACCAA
HSV-1 UL30 (Pol)	CGAGTGCGAAAAGACGTTCA	TGGAGGTGCGGTTGATAAAC
HSV-1 UL27 (gB)	CTGCGAAACGGTGACGTCTT	GCCCGAGGGTCAGAACTACA
HSV-1 UL44 (gC)	TCCACCCTGCCCATTTCGTA	GCGAGAACCCCGATTCCAAT
HSV-1 UL49 (VP22)	GGTATGGCGAGTCCCGATAG	GTTCGTCGTCTTCGGATGAC
SeV NP	TGCCCTGGAAGATGAGTTAG	GCCTGTTGGTTTGTGGTAAG
NDV HN	CAATGCTGAGACAATAGGTC	GACAATGCTTGATGGTGAAC
Hu-IFN-β	GCCAGCGTCTGACGTTATGA	GAGGGCTGATCCTACCACAA
Hu-IRF7	GGTGTGTCTTCCCTGGATAG	GCTCCAGCTCCATAAGGAAG
Hu-IFN-γ Receptor 2	ACCCTCCAGAGTGTACTGTT	AGCACCGACAGCAACGAAAA
Hu-IL-12 Receptor B	ACTGGAGCCTCAGCACATCT	AGAGCATGAGGGAGTCACAC
Hu-β-actin	ACTGGAACGGTGAAGGTGAC	GTGGACTTGGGAGAGGACTG
Hu-α-tubulin	GCCTGGACCACAAGTTTGAC	TGAAATTCTGGGAGCATGAC
Hu-rps11	GCCGAGACTATCTGCACTAC	ATGTCCAGCCTCAGAACTTC
Mu-IFN-β	AGATGTCCTCAACTGCTCTC	AGATTCACTACCAGTCCCAG
Mu-IFN-α	TCCTGAGCCAAAGTGTAGAG	GAGAACAAGTGCCTTTACAG
Mu-IL-6	ACAGAAGGAGTGGCTAAGGA	CGCACTAGGTTTGCCGAGTA
Mu-IL-12p40	TTGAAAGGCTGGGTATCGGT	GAATTTCTGTGTGGCACTGG
Mu-β-actin	AGGTGACAGCATTGCTTCTG	GCTGCCTCAACACCTCAAC
Mu-rps11	CGTGACGAAGATGAAGATGC	GCACATTGAATCGCACAGTC

### Statistical Analysis

Statistical analysis was performed using *t* test, paired two sample analysis.

## Results

### HSV-1 Activates cDCs through Replication-Dependent and -Independent Pathways Both of Which Are Inhibited by vhs

HSV-1 viruses carrying mutations in the gene encoding the vhs protein, U_L_41, display a relatively minor reduction in the ability to replicate in most transformed cell lines [26], [27], [39]. However, vhs disruptions produce a much more dramatic growth defect in whole animal models [40], [41], [42]. We first evaluated the capacity of a recombinant vhs-deficient HSV virus (vhs^−^) [27] to infect and replicate in primary human monocyte-derived conventional DCs (hu-cDCs). Control hu-cDCs were mock-infected or infected with the HSV-1 wild type parent strain KOS. Virus released into the media was quantified by plaque assay at different times post infection (hpi) (Figure 1a). As expected, both HSV-1 and the vhs- virus infected monocyte-derived hu-cDCs productively and replicated to low levels, which is consistent with the work of others [10]. Notably, there was an approximately 60-fold difference between the amount of infectious virus released by vhs–deleted and KOS-infected DCs at the 48 hour time-point. The relative expression of the immediate early gene RL2 (ICP0) was only modestly affected (2.2-fold) by the absence of vhs at 6 hpi but it was significantly reduced (30-fold) by 18 hpi (Figure 1b). We noted a similar difference for multiple HSV-1 genes over this time course (Figure 1c). We further characterized the infection of hu-cDCs by HSV-1 using an HSV-GFP virus (Figure S1a). At an MOI of 5 (utilized in experiments throughout this study), we noted GFP expression in the majority of cells. Additionally, as shown in Figure S1b, quantitation of the major capsid protein VP5 was similar for KOS, vhs-, and GFP infections showing that incoming virus is similar for all different virus treatments utilized.

**Figure 2:**
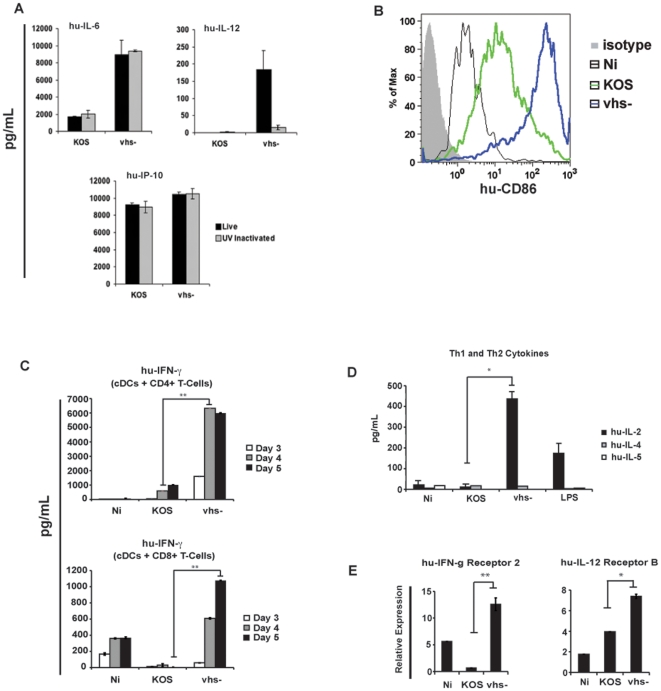
Virion-associated vhs blocks the activation of cDCs and can influence the phenotype of CD4+ T-cells during allogeneic *in vitro* co-culture. (a.) hu-cDCs were infected with infectious (live) and UV inactivated KOS or vhs- at an MOI of 5. At 24 hpi, media from cultures were collected and assayed for secreted IL-6, IL-12p70, and IP-10 using multiplex ELISA. Error bars represent the difference between duplicate assays. (b.) hu-cDCs were infected with KOS and vhs- viruses at an MOI of 5; at 24 hpi, cells were harvested, and cell surface expression of hu-CD86 was measured by Flow Cytometry. (c.) hu-cDCs were infected with KOS and vhs- viruses at an MOI of 5. After a 1-hour infection, virus was removed and the hu-cDCs were co-cultured in a 1∶5 ratio with either naïve CD4+ or CD8+ T-cells. At days 3, 4, and 5, cells were harvested and the supernatants were analyzed for secreted IFN-γ by ELISA. Error bars represent the difference between duplicate assays. ** denotes a p-value <0.005. (d.) Co-cultures of cDCs and CD4+ T-cells were harvested at day 4 and assayed for secreted IL-2 (Th1 cytokine), IL-4 and IL-5 (Th2 cytokines) by Multiplex ELISA. * denotes a p-value <0.05 (e). Co-cultures of cDCs and CD4+ T-cells were harvested at day 4. RNA was isolated from cell pellets and subject to qRT-PCR to analyze the relative expression of Th1-associated genes relative to normalized housekeeping genes. * denotes a p-value <0.05; ** denotes a p-value <0.005.

Consistent with a prior study using a highly mutated replication-defective HSV virus, the induction of pro-inflammatory cytokines (IL-6 and TNF-α) in response to HSV-1 was blocked by vhs (Figures 2a and S3) [31]. Interestingly, the vhs protein inhibited both the virus replication independent triggering of IL-6 and TNF-α (UV inactivated virus) and the replication dependent induction of IL-12, highlighting that the activation of cDCs in response to HSV-1 occurs after sensing viral replication dependent and independent events. The production of the chemokine IP-10 was not affected by vhs. A detailed kinetic analysis revealed that the block to IL-6 and TNF-α production occurs as early as 6 hpi, whereas IL-12 release is blocked by 12 hpi (Figure S2b). This block to cytokine release is also observed at the level of mRNA expression (Figure S2c). Lastly, we also observed elevated cell surface expression of the co-stimulatory molecule CD86 in the vhs- infected cultures at 24 hpi (Figure 2b).

### Vhs Influences the Phenotype of T-Cells upon *In Vitro* Allogeneic Co-Culture with Productively Infected cDCs

HSV-1 infection results in the polarization of CD4+ T-cells towards a Th1 response characterized by the production of IFN-γ and IL-2 and the absence of Th2 cytokines such as IL-4 and IL-5 [43], [44], [45], [46]. The capacity for hu-cDCs productively infected with KOS or vhs-deficient viruses to stimulate T-cells was assessed using an allogeneic co-culture assay. Both CD4+ and CD8+ T-cells produced significantly more IFN-γ upon co-culture with cDCs infected with the vhs-deficient virus than with wild-type KOS virus (Figure 2c). The decrease in IFN-γ for KOS infection in the CD8+ co-culture is consistent with HSV-1 actively blocking allogeneic stimulation. In addition, CD4+ T-cell co-cultures containing cDCs infected with the vhs- virus produced more IL-2 than cultures with KOS infected cDCs (Figure 2d). IL-4 or IL-5 were not detected in these cultures. Analysis of gene expression by RT-PCR revealed enhanced up-regulation of other genes associated with a Th1 response when stimulated with vhs- infected cDCs relative to KOS infected cDCs (Figure 2e). Taken together, these data show that during a productive HSV-1 infection vhs negatively regulates the development of Th1 immunity.

### Vhs Does Not Block the Activation of Plasmacytoid DCs

**Figure 3:**
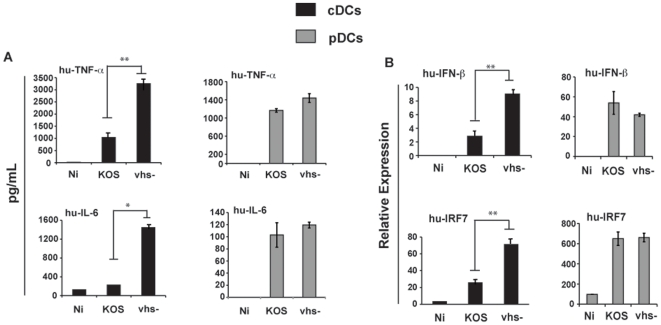
Vhs does not block pDC cytokine secretion. (a.) Plasmacytoid DCs (hu-pDCs) were directly isolated from buffy coats and infected with KOS and vhs- viruses at an MOI of 5. Hu-cDCs were cultured from monocytes isolated from the same buffy coats and infected similar to hu-pDCs. At 6 hpi, supernatants were analyzed for secreted TNF-α and IL-6 by ELISA. Error bars represent the difference between duplicate assays. (b.) RNA was extracted from cell pellets at the 6 hour time-point, reverse-transcribed and subject to qRT-PCR for relative expression of IFN-β and IRF7 to normalized housekeeping genes. * denotes a p-value <0.05; ** denotes a p-value <0.005.

Plasmacytoid DCs (pDCs) represent a class of DCs specialized in the production of type I IFNs in response to virus infection [47]. To compare the effect of vhs during HSV-1 infection of pDCs to that observed with cDCs, both cell types were prepared from the same donor and infected with vhs- and KOS viruses. As shown in Figure 3a, the vhs-mediated inhibition of IL-6 and TNF-α production by cDCs is evident at 6 hpi. In contrast, vhs is unable to alter the production of these cytokines by pDCs. Similar results were also observed at the level of mRNA expression (Figure S4d). There were no significant differences in measurements of transcription of the IFN-β and IRF7 genes (an IFN-responsive gene) following infection of pDCs with vhs- and KOS viruses.

We observed slightly less GFP expression following infection of pDCs with HSV-GFP compared to cDCs (Figure S4a). Similarly, we also detected less expression of the immediate early transcript UL54 in comparing infections between the two cell types. (Figure S4b). To ensure that the observed phenotypic difference between cDCs and pDCs was not simply due to a less efficient infection of the pDC, we increased the input of virus by 2- and 10-fold. As shown in Figure S4c, even at high MOIs, there is no significant difference in the cytokine response comparing infection of pDC with wild type virus to the vhs-deficient virus. We conclude that the negative regulatory role of vhs is functional in cDCs but not pDCs.

### HSV-1 Modulates Murine DC Activation Similar to Human DC Activation

The pDC response to HSV-1 has been reported to occur in a TLR9-dependent fashion [16], [48]. In contrast, while cDCs express TLRs, they have also been shown to respond to viral infection by signaling through cytosolic pathways (RIG-I and mda5), which recognize RNA molecules [47]. Also noteworthy is the recent finding that a cytoplasmic DNA sensor is responsible for recognition of DNA viruses and subsequent production of type I IFN *in vitro* [49]. The failure of vhs to block the activation of pDCs led us to speculate that inhibition of cDC cytokine release by vhs may occur through a pathway independent of TLR signaling.

**Figure 4:**
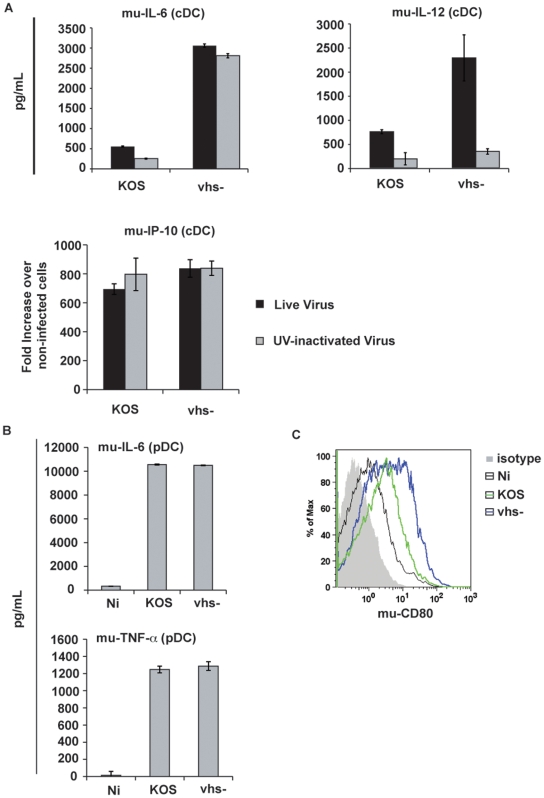
HSV-1 modulates the activation of murine-derived DCs similar to human-derived DCs. (a.) mu-cDCs were infected with both live and UV inactivated virus stocks at an MOI of 5. At 12 hpi, cells were harvested, and the levels of IL-6 and IL-12p40 were measured in the supernatants (ELISA). qRT-PCR was used to measure the mRNA levels of IP-10 within infected cells. Data is represented as relative expression relative to levels of housekeeping genes. Error bars denote the difference between duplicate (ELISA) and triplicate (PCR) assays. (b.) mu-pDCs were infected with KOS and vhs- viruses at an MOI of 5; released IL-6 and TNF-α were measured by ELISA assay at 6 hpi. (c.) mu-cDCs were infected with KOS and vhs- viruses; cell surface expression of mu-CD80 was measured by Flow Cytometry at 24 hpi.

Due to the inherent difficulties in genetically manipulating human systems in addressing mechanistic questions, we shifted our study to a mouse bone marrow-derived DC system. As shown in Figure 4, HSV-1 modulated the secretion of pro-inflammatory cytokines and co-stimulatory molecules in murine DCs in a manner similar to that of human DCs (Figure 2a, 2b and Figure 3a). We also detected the expression of HSV-1 lytic transcripts during infection mu-cDCs (data not shown).

### HSV-1 Activation of mu-cDCs Does Not Depend on TLR Signaling and Is Blocked by the vhs Protein

**Figure 5:**
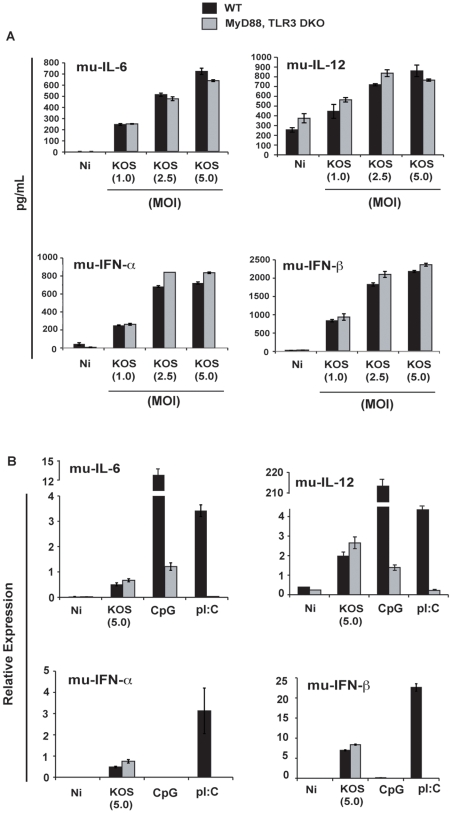
DCs are activated in a TLR-independent manner during HSV-1 infection. (a.) mu-cDCs isolated from MyD88, TLR3 double knock-out (DKO) and control C57BL/6 wild-type mice were infected with increasing amounts of wild-type HSV-1 (KOS). At 12 hpi, cells were harvested and released IL-6, IL-12p40, IFN-α, and IFN-β were quantitated by ELISA. (b.) RNA was extracted from the cell pellets from the above experiment and subject to qRT-PCR for the corresponding genes. Data is represented as relative expression relative to levels of housekeeping genes. CpG (6 ug/mL) was utilized to control for and confirm MyD88 deficiency; pI:C (250 ug/mL) was utilized to control for and confirm TLR3 deficiency.

To evaluate the role of TLR signaling during HSV-1 infection of cDCs, we first infected mu-cDCs isolated from double knock-out mice deficient in both MyD88 and TLR3 with increasing amounts of wild-type HSV-1 (KOS). As shown in Figure 5a, HSV-1-infected mu-cDCs secreted pro-inflammatory cytokines (IL-6 and IL-12) and type I interferons (IFN-α and IFN-β) independently of TLR signaling and in a dose-dependent manner. This effect was also seen at the transcriptional level for KOS infection at an MOI of 5.0 (Figure 5b).

**Figure 6:**
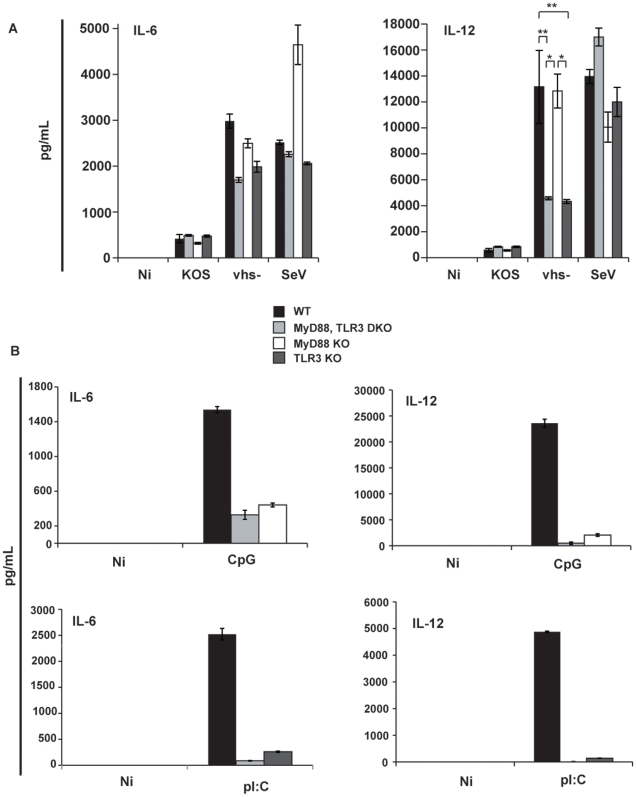
Vhs blocks a TLR-independent pathway of DC activation during HSV-1 infection. (a.) mu-cDCs isolated from MyD88, TLR3 double knock-out (DKO), MyD88 KO, and TLR3 KO mice were infected with KOS, vhs-, and SeV at an MOI of 5 for HSV-1 and 1.5 for SeV. Mu-cDCs from control C57BL/6 mice were isolated on three separate occasions and infected with virus. (b.) CpG (6 ug/mL) and pI:C (250 ug/mL) were used to control for and confirm MyD88 and TLR3 deficiencies, respectively. IL-6 and IL-12p40 levels were measured in the supernatants of infected cultures at 12 hpi. For knock-out infections, error bars denote the difference between duplicate assays. For wild-type (C57BL/6) infections, error bars denote the difference between 3 independent experiments. * denotes a p-value <0.05; ** denotes a p-value <0.005.

Infection of mu-cDCs with the vhs-deficient virus triggered significantly higher cytokine release than the KOS virus. This was observed with mu-cDCs isolated from either control wild-type mice, MyD88/TLR3 double knock-out, MyD88 single knock-out, or TLR3 single knock-out mice showing that vhs can block the secretion of cytokines even in cDCs derived from animals incapable of TLR signaling (Figure 6a). As a control, we infected all mu-cDCs with Sendai virus, which our group has previously shown to activate DCs independent of the TLR pathway [50]. Interestingly, upon comparing the secretion of IL-12 from the three different knock-out conditions during vhs- infection it was apparent that the presence of TLR3 significantly augments the response to the highly stimulatory vhs- virus. Figure 6b represents the assay controls.

### Vhs Blocks the Activation of hu-cDCs and mu-cDCs by SeV and NDV, but Not by TLR Agonists

**Figure 7:**
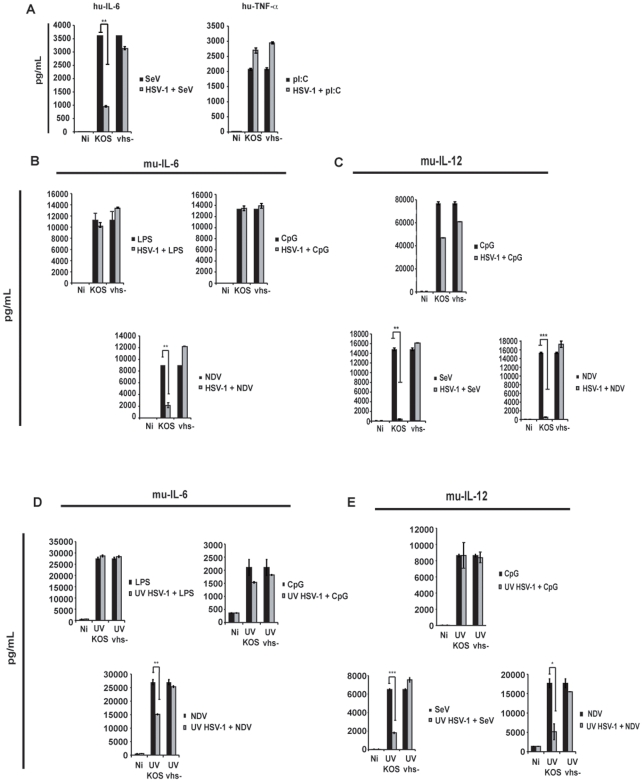
Vhs fails to block the activation of DCs due to TLR agonists. (a.) hu-cDCs were infected with either SeV at an MOI of 1.5 or treated with pI:C (250 ug/mL) for 6 hpi [black bars]; In parallel, hu-cDCs were infected with KOS and vhs- at an MOI of 5 and then immediately treated with SeV or pI:C [gray bars]. At 6 hpi, released IL-6 and TNF-α were measured in the supernatants of cells. (b.) mu-cDCs were treated with LPS (300 ng/mL), CpG (6 ug/mL), or infected with NDV (MOI of 1.5) for 6 hpi [black bars]; In parallel, mu-cDCs were infected with KOS and vhs- at an MOI of 5 and then immediately treated with LPS, CpG, or NDV [gray bars]. At 6 hpi, released IL-6 was measured in the supernatants of cells. (c.) Same set-up as in (b.). IL-12p40 was measured in the supernatants of cells at 6 hpi. (d. and e.) Identical set-up to (b and c.), this time infections were performed using UV-inactivated HSV-1 virus stocks. Error bars denote the difference between duplicate assays. * denotes a p-value <0.05; ** denotes a p-value <0.005; *** denotes a p-value <0.0005.

To expand on the findings using TLR knock-out mice we infected human and mouse cDCs (Figure 7) with HSV-1 (either KOS or vhs-) and immediately co-treated the cells with either TLR agonists (pI:C, LPS, or CpG) or RIG-I/mda5 agonists (SeV and NDV). While infection with vhs- virus resulted in the production of cytokines in response to co-treatment with TLR agonists or virus, infection with wild type virus resulted in impaired production of cytokines in response to either SeV or NDV, but not to TLR ligands. This capacity for vhs to block SeV and NDV activation of the cDC was significant even in the absence of virus replication at the 6 hpi time-point (Figure 7c).

These results indicate that vhs inhibits the TLR-independent activation of DCs but not the TLR-dependent response. These observations are consistent with both the inability of vhs to block the TLR-dependent pDC response to HSV (Figure 3), as well as the TLR-independent antagonistic nature of this protein (Figure 6).

### Vhs Enhances the Ability of SeV and NDV to Replicate in mu-cDCs

**Figure 8:**
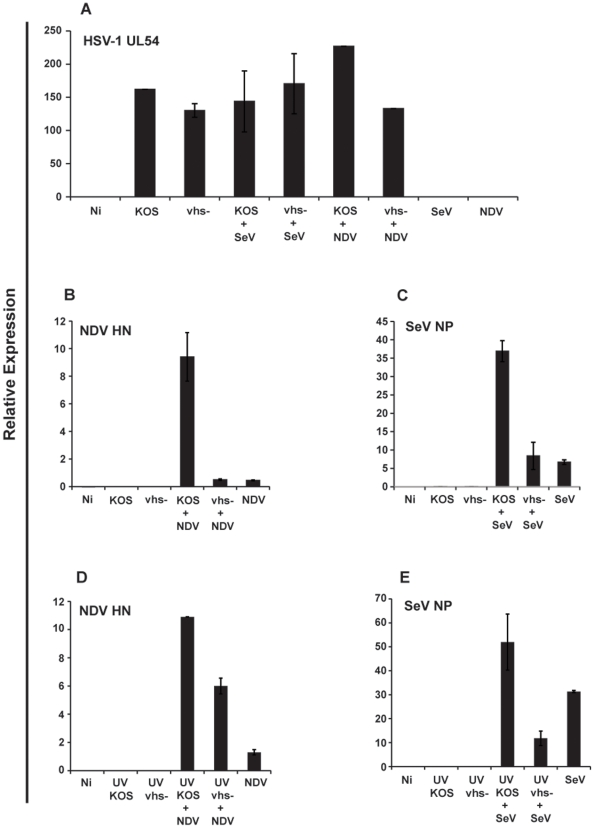
Higher expression of SeV and NDV specific genes during wild-type HSV-1 infection. RNA was extracted from the cell pellets of mu-cDCs following co-infections/co-treatments with TLR agonists at 6 hpi. qRT-PCR was used to check expression levels for the following viral genes: (a.) UL54 (HSV-1), (b. and d.) HN (NDV) and (c. and e.) NP (SeV). Data is represented as relative expression relative to levels of housekeeping genes. Error bars denote the difference between triplicate assays.

Based on the ability of vhs to block both SeV and NDV-induced cytokine production in mu-cDCs, we hypothesized that expression of viral genes specific to these viruses would be elevated during co-infections with KOS compared to vhs-. As shown in Figure 8, expression of the NP gene of SeV and the HN gene of NDV are both elevated when co-infected with the vhs-containing wild-type KOS relative to the vhs- co-infection for both live and UV inactivated viruses. Thus, infection with HSV makes cells more susceptible to other infectious agents through the action of the vhs protein.

## Discussion

Our results demonstrate that during a productive HSV-1 infection the vhs protein carried in the tegument can inhibit the early innate immune response in both human and mouse monocyte-derived cDCs. These data suggest that this protein, released into infected cells at the time of infection, inhibits the triggering of cDCs probably until other antagonistic proteins are produced. HSV is capable of productively replicating in cDCs, and its replicative capacity is improved by the presence of the vhs protein (Figure 1). This observation is presumably due to a synergism of 2 events: 1.) inefficient immediate early gene expression in the absence of vhs (Figure 1b and 1c) and 2.) elevated type I IFN found in vhs- infected cells (Figure 3b). A previous report showed that infection of cDCs with a replication-defective viral construct lacking vhs resulted in elevated levels of pro-inflammatory cytokines at late time-points post infection compared to the same viral construct containing vhs [31]. Since we hypothesized that vhs functions early in infection we analyzed the cellular response at early times points. We confirmed that there was an enhanced release of IL-6 and TNF-α in dendritic cells in response to UV-inactivated virus lacking vhs. Interestingly, IL-12 production by infected DCs was also susceptible to vhs inhibition but was only produced in response to live virus. These data indicate that cDC activation during HSV-1 infection is triggered through both replication-dependent and replication-independent pathways. In addition, experiments using T cell co-cultures demonstrated that the vhs protein impaired the development of Th1 immunity as shown by the impairment in the expression of a number of Th1-related genes. These results demonstrated a critical role of the vhs protein in controlling the development of both innate and adaptive immunity in response to HSV-1 infection.

An important observation derived from our studies is that the viral vhs protein inhibits a TLR-independent pathway of cell activation (Figure 6). A number of studies have shown that TLR9 is utilized in the recognition of HSV-1 by pDCs [16], [48] while the response to HSV-1 in cDCs is independent of TLR2, TLR9 and MyD88 [16], [51]. The role of TLR3 in the DC response to HSV is not well established. We found that infection of mu-cDCs isolated from mice double deficient in MyD88 and TLR3 with the KOS strain of HSV-1 responded identically to mu-cDCs from wild-type control mice (Figure 5a). Thus, cDCs are capable of responding to the KOS 1.1 strain of HSV-1 in the absence of any of the TLRs typically attributed to viral recognition (TLR2, 3, 7, 8, and 9). In addition, vhs fails to block the secretion of IL-6 from LPS-treated (TLR4 stimulated) mu-cDCs upon co-treatment with HSV-1 (Figure 7b and 7d) again suggesting that the vhs antagonism is functional against a pathway not triggered through a TLR. The inability of vhs to block LPS-stimulated DCs in our study is in contrast to a previous report showing a repression of co-stimulatory molecule up-regulation upon infection with a viral construct containing vhs [31]. One potential explanation for this difference is that the ability of vhs to antagonize NF-kB-dependent genes (IL-6, TNF-α) may be separate from its ability to block IFN-dependent events (co-stimulatory molecule up-regulation).

Interestingly, the secretion of IL-12 following infection with the highly stimulatory vhs-deletion virus was enhanced when TLR3 was present (Figure 6a). Although we observed 10-fold higher levels of IL-12 in the vhs- infections relative to KOS for cells isolated from mice deficient in TLR3, we suspect that the difference compared to infection of mu-cDCs from wild-type mice and MyD88 single knock-out mice (where TLR3 is present and functional) may be attributable to the function of vhs as a viral nuclease (see below). During cDC infection with the highly stimulatory vhs- virus, dsRNA molecules may accumulate due to a failure of vhs to efficiently degrade viral replication intermediates [52]. Such viral intermediates could potentially activate DCs through TLR3 and enhance the signaling.

Our results are consistent with vhs blocking a viral sensing pathway independent of TLRs. Despite observing elevated mRNA expression of pro-inflammatory cytokines and type I IFN in the vhs- infected cDC cultures (Figures S2c and 3), we failed to find such differences in the co-treatment experiments when HSV-1 infected DCs were stimulated with TLR agonists (Figure 7). This suggests that the target(s) of vhs is not these mRNA transcripts, but rather at some point upstream of their expression. Specifically, the activation step for both HSV-1 as well as the paramyxoviruses SeV and NDV appear to be blocked by vhs. Both SeV and NDV induced activation of hu- and mu-cDCs including pro-inflammatory cytokine release. However, this activation was blocked when the cells were co-infected with KOS. This is consistent with an earlier report showing that HSV-1 blocked type I Interferon production by SeV [53]. We further report here that removal of vhs relieves this block to DC cytokine release observed during co-infection with KOS and either SeV or NDV. DC activation due to the paramyxoviruses SeV and NDV has been reported to occur by signaling through the intracellular receptors RIG-I and mda5 and to be dependent upon virus replication [54], [55]. In addition, it has been recently shown that RIG-I contributes to the recognition of HSV-2 in macrophages [19]. Our results argue that vhs may actively block signaling due to either RIG-I, mda5, or both. Experiments to investigate what role these cytoplasmic sensors play in the vhs-mediated inhibition of cytokines during HSV-1 infection are underway.

The observation that infection with one pathogen can enhance the susceptibility to another, or alter its disease progression, has been reported for multiple viruses [56], [57], [58]. Understanding which viral factors are responsible for such observations may be important for the optimal design of therapeutics and/or vaccines. With respect to HSV, interest in vhs-deletion vaccine candidates has previously been reported [59]. The presence of the vhs protein fosters a cellular environment optimal for the replication of HSV-1 during a productive infection (Figure 1). If the promotion of viral replication results from the inhibition of pro-inflammatory cytokines and type I IFN by vhs in cDCs (Figures 2 and 3), then HSV co-infection might create a milieu conducive to infection by other viruses. We observed both a repression of cytokine release (Figure 7) and an enhancement of their respective viral gene expression (Figure 8) when SeV or NDV were co-infected with KOS. We noted a similar result when UV-inactivated viruses were utilized in these co-treatment experiments. However, while still significant, there were elevated levels of both IL-6 and IL-12 when SeV and NDV were co-infected with UV KOS (Figure 7d and e). This suggests that the influence of vhs on the expression of other HSV-1 genes (Figure 1c) may contribute to the strong inhibition observed during live virus co-infections (Figure 7b and c). The data presented here shows that the vhs protein is at least partially responsible for this effect.

Vhs is a nuclease, which belongs to a family of endonucleases that includes DNAse IV, ERCC-5, and yeast RAD2 [60], [61], [62]. It acts to degrade cellular and viral mRNAs early in HSV infection. Although vhs was originally thought to act globally to limit all gene expression, recent reports have determined that some cellular mRNA molecules are resistant to vhs activity during infection [63], [64]. Consistent with this data, our results indicated that the levels of the chemokine IP-10 are not affected by vhs (Figure 2 and 4). Interestingly, when we compared the activity of vhs in cDCs to MEFs (mouse embryo fibroblast cells), we observed that cDCs appear to be more resistant to vhs as measured by Ct levels of cellular housekeeping genes (Figure S5), again suggesting that the effect of vhs is not simply global degradation. Other reports have shown that neurons are less affected by the vhs protein [65], [66]. This phenotype was shown to be dependent on the amount of input virus [66]. An analysis of additional cell types, dose-response experiments using our viruses, and other assays to assess vhs activity are in progress. It is also possible that the inhibition of DC maturation is occurring through an as yet unidentified function of vhs. A recent report has shown that a portion of vhs is associated with lipid rafts in cellular membranes [67]. The lipid-associated vhs would be in the correct location to interact with cellular molecules involved in signal transduction. Mutational analysis of the vhs protein would allow us to determine whether the nuclease function of vhs can be separated from its effect on DC activation.

HSV-1 primarily infects epithelial cells [2] and cDCs residing in the epithelial tissue. The viral vhs protein may have evolved to silence the pro-inflammatory response induced in these cells to allow the replication of its genome and help secure its latent phenotype in neurons. Our data demonstrate that vhs does not influence the expression and secretion of pro-inflammatory cytokines and type I IFN by pDCs. These cells do not reside in epithelial tissues [68] and may not be exposed to the virus early in the primary infection. Moreover, a recent study concludes that cDCs are crucial for presentation to CD4^+^ and CD8^+^ T-cells but pDCs are not involved in the activation of T-cells during HSV-1 infection [69]. Thus, the inhibition of cDC maturation (Figures 2a and 3) but not pDCs and the enhanced ability of cDC infected with the vhs-deletion virus to stimulate T-cells (Figure 2b) would seem to be correctly targeted to suppress T cell activation.

In summary, the results of these studies have demonstrated that vhs carried in the tegument is a potent early antagonist of DC activation during a productive HSV-1 infection. Further, vhs blocks a pathway to cytokine release in cDCs that is independent of TLR signaling. These insights into DC function during infection may be valuable to herpes-based vaccine development and gene therapy research.

## Supporting Information

Figure S1 (a.) GFP expression in hu-cDCs. Hu-cDCs were infected with HSV-GFP at an MOI of 5. At 18 hpi, cells were harvested and GFP was measured by Flow Cytometry; analysis was performed using Flowjo. (b and c.) Incoming VP5 expression. Hu-cDCs were infected with KOS, vhs-, and HSV-GFP at an MOI of 5. At 3 hpi, cells were harvested, fixed in 0.5% PFA, permeabilized in 0.1% Saponin, and stained for intracellular expression of the major HSV-1 capsid protein VP5. Measurement of internalized VP5 was performed using Flow Cytometry; analysis was conducted using Flowjo. A graphical representation is shown in (c.)
(1.03 MB PDF)

Figure S2 (a.) Mock Vero Cell Lysate does not activate hu-cDCs. Hu-cDCs were infected with KOS and vhs- virus at an MOI of 5. Mock Vero lysate and New Castle Disease Virus (NDV) infections were used as controls. At 18 hpi, the media from the cultures was collected and assayed for secreted IL-6 and TNF-Î± using multiplex ELISA. Error bars represent the difference between duplicate assays. (b.) Vhs blocks the activation of cDCs by 6 hpi for IL-6 and TNF-Î±; by 12 hpi for IL-12p70. Hu-cDCs were infected with KOS and vhs- viruses at MOIs of 0.5 or 5. Media was collected at various times post infection and analyzed for the presence of secreted IL-6, TNF-Î±, IL-12p70, and IP-10 using multiplex ELISA. Error bars represent the difference between duplicate assays. (c.) Higher mRNA expression of pro-inflammatory cyokines in vhs- infected hu-cDC cultures. Hu-cDCs were infected with KOS and vhs- viruses at an MOI of 5. At 6 and 12 hpi, cells were harvested; RNA isolated and subject to qRT-PCR looking at the relative expression of IL-6, TNF-Î±, and IL-12p40.
(2.89 MB PDF)

Figure S3 Stocks of KOS and vhs- virus were exposed to UV irradiation and used to infect hu-cDCs at volumes equivalent to MOIs of 0.05, 0.5, and 5. At 24 hpi, media from cultures were collected and analyzed for secreted TNF-Î± and IL-6 using multiplex ELISA. Error bars represent the difference between duplicate assays.
(0.16 MB PDF)

Figure S4 (a) GFP expression in hu-pDCs. Hu-pDCs were infected with HSV-GFP, harvested at 12 hpi, and subject to Flow Cytometry to measure GFP expression; analysis was performed using Flowjo. (b.) Comparing HSV-1 gene expression in hu-cDCs to hu-pDCs. Monocyte-derived human dendritic cells (hu-cDCs) and plasmacytoid dendritic cells (hu-pDCs) isolated from the same donor were infected with HSV-1 at an MOI of 5. At 6 hpi, cells were harvested, RNA isolated and subject to qRT-PCR looking at the relative expression of the HSV-1 viral gene UL54. Error bars represent the difference between triplicate assays. (c.) Dose-response HSV-1 infection of hu-pDCs. Hu-pDCs were infected with KOS and vhs- viruses at MOIs of 10 and 50; Cells were harvested at 6 hpi and subject to ELISA assay for released IL-6 and TNF-Î±. Error bars represent the difference between duplicate assays. (d.) Pro-inflammatory cytokine mRNA levels are negatively regulated by vhs in cDCs, but not in pDCs. Same as in (b.), qRT-PCR was used to measure relative gene expression of IL-6, TNF-Î±, and IL-12p40. Error bars represent the difference between triplicate assays.
(1.06 MB PDF)

Figure S5 Hu-cDCs and Mouse Embryo Fibroblast (MEF) cells were infected with KOS and vhs- viruses at an MOI of 5. At 18 hpi, cells were harvested, RNA was isolated and subjected qRT-PCR. Levels of the housekeeping genes rps11 and β-actin were measured. Data shown is a representative from multiple experiments and presented as median Ct value. Error bars represent the difference between triplicate assays.
(1.00 MB PDF)
