# Supplementary figures and images for: The Virion Host Shut-Off (vhs) Protein Blocks a TLR-Independent Pathway of Herpes Simplex Virus Type 1 Recognition in Human and Mouse Dendritic Cells
Source: PLoS One. 2010 Feb 18;5(2):e8684. doi: 10.1371/journal.pone.0008684 (PMC2823768; doi:10.1371/journal.pone.0008684)

Supplementary Figure #1

A

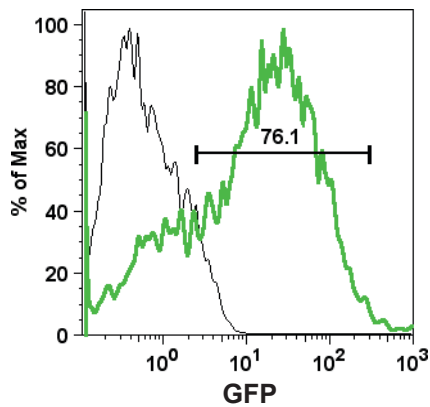

B

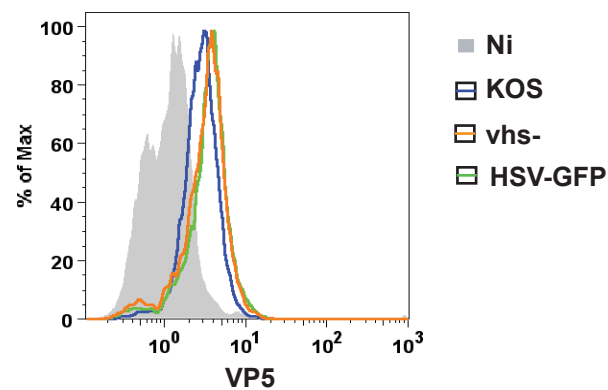

C

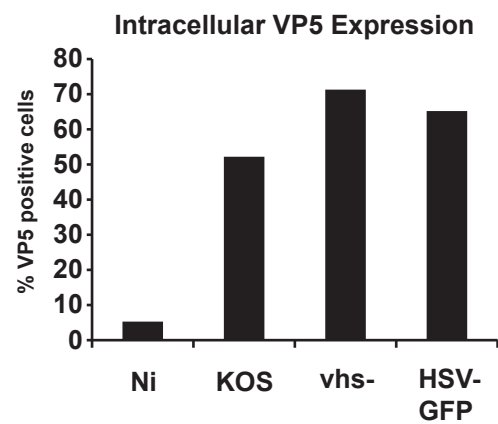

Supplement: Figure S1 — (a.) GFP expression in hu-cDCs. Hu-cDCs were infected with HSV-GFP at an MOI of 5. At 18 hpi, cells were harvested and GFP was measured by Flow Cytometry; analysis was performed using Flowjo. (b and c.) Incoming VP5 expression. Hu-cDCs were infected with KOS, vhs-, and HSV-GFP at an MOI of 5. At 3 hpi, cells were harvested, fixed in 0.5% PFA, permeabilized in 0.1% Saponin, and stained for intracellular expression of the major HSV-1 capsid protein VP5. Measurement of internalized VP5 was performed using Flow Cytometry; analysis was conducted using Flowjo. A graphical representation is shown in (c.) (1.03 MB PDF) [file pone.0008684.s001.pdf]

Supplementary Figure 2

A

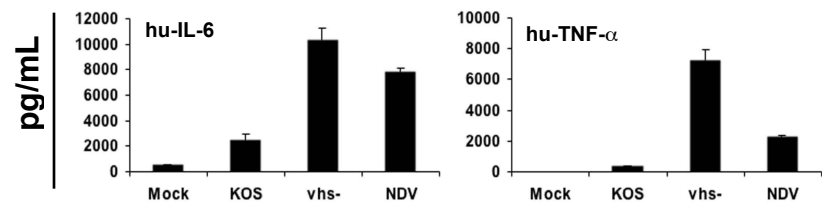

B

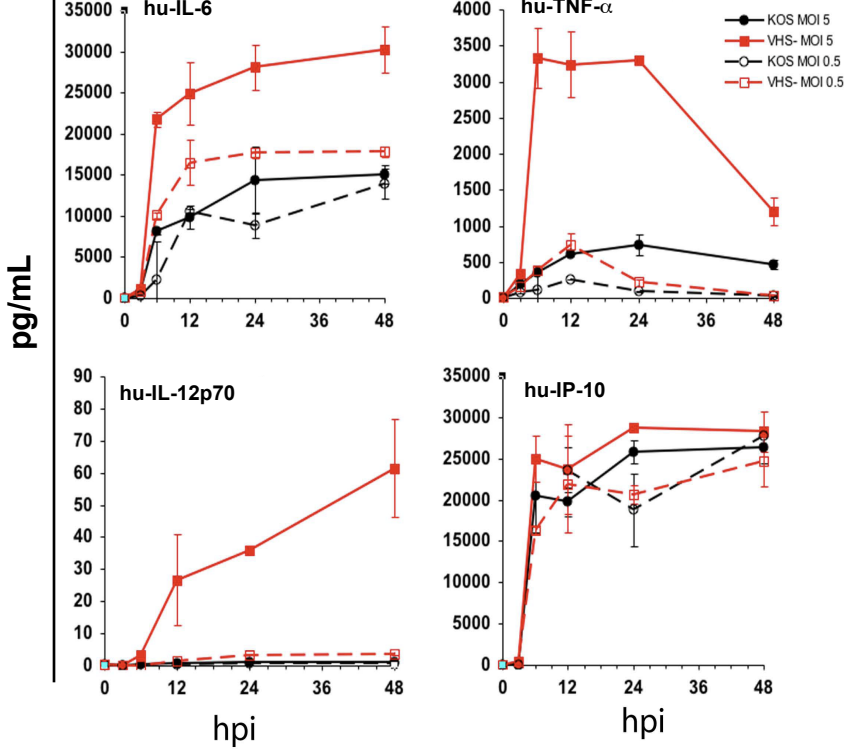

C

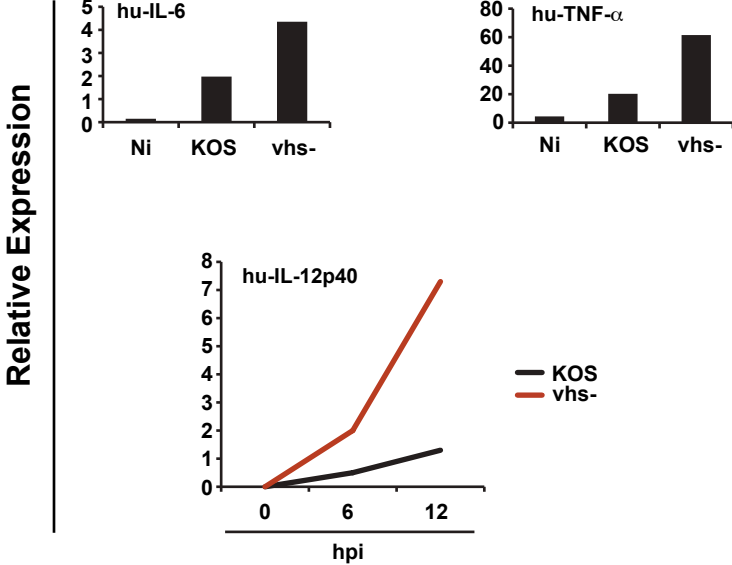

Supplement: Figure S2 — (a.) Mock Vero Cell Lysate does not activate hu-cDCs. Hu-cDCs were infected with KOS and vhs- virus at an MOI of 5. Mock Vero lysate and New Castle Disease Virus (NDV) infections were used as controls. At 18 hpi, the media from the cultures was collected and assayed for secreted IL-6 and TNF-Î± using multiplex ELISA. Error bars represent the difference between duplicate assays. (b.) Vhs blocks the activation of cDCs by 6 hpi for IL-6 and TNF-Î±; by 12 hpi for IL-12p70. Hu-cDCs were infected with KOS and vhs- viruses at MOIs of 0.5 or 5. Media was collected at various times post infection and analyzed for the presence of secreted IL-6, TNF-Î±, IL-12p70, and IP-10 using multiplex ELISA. Error bars represent the difference between duplicate assays. (c.) Higher mRNA expression of pro-inflammatory cyokines in vhs- infected hu-cDC cultures. Hu-cDCs were infected with KOS and vhs- viruses at an MOI of 5. At 6 and 12 hpi, cells were harvested; RNA isolated and subject to qRT-PCR looking at the relative expression of IL-6, TNF-Î±, and IL-12p40. (2.89 MB PDF) [file pone.0008684.s002.pdf]

Supplementary Figure 3

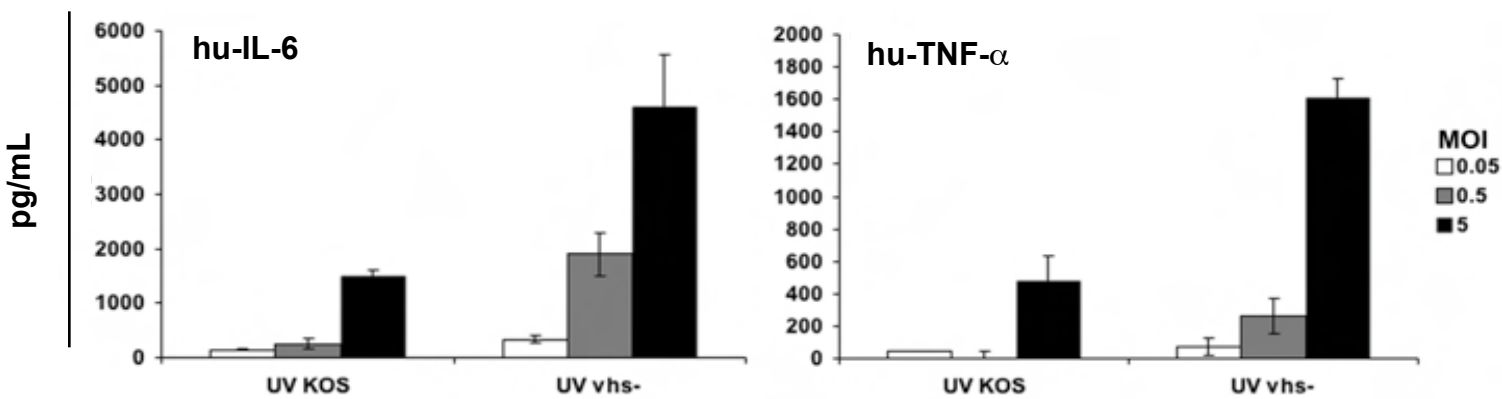

Supplement: Figure S3 — Stocks of KOS and vhs- virus were exposed to UV irradiation and used to infect hu-cDCs at volumes equivalent to MOIs of 0.05, 0.5, and 5. At 24 hpi, media from cultures were collected and analyzed for secreted TNF-Î± and IL-6 using multiplex ELISA. Error bars represent the difference between duplicate assays. (0.16 MB PDF) [file pone.0008684.s003.pdf]

Supplementary Fig. 4

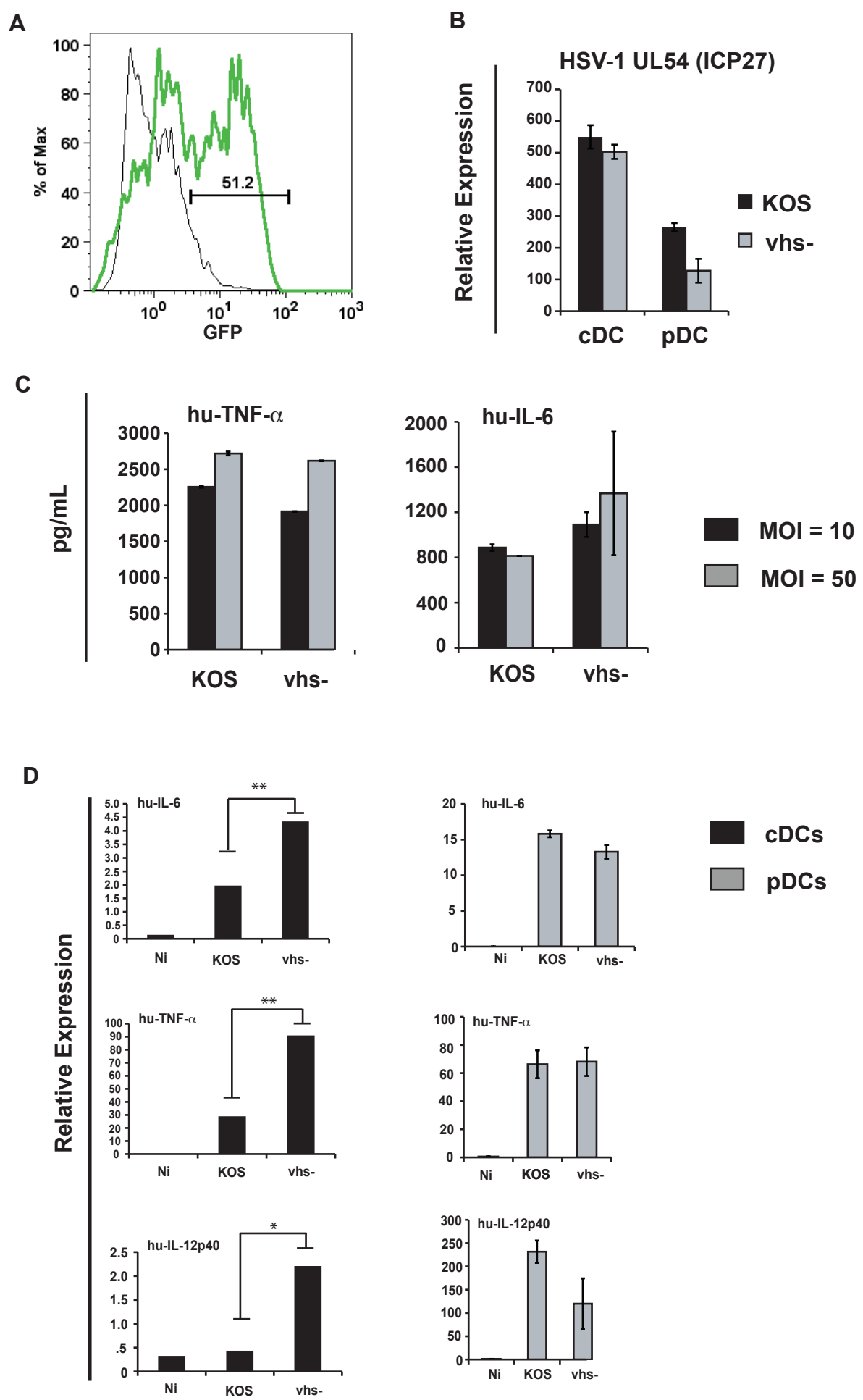

Supplement: Figure S4 — (a) GFP expression in hu-pDCs. Hu-pDCs were infected with HSV-GFP, harvested at 12 hpi, and subject to Flow Cytometry to measure GFP expression; analysis was performed using Flowjo. (b.) Comparing HSV-1 gene expression in hu-cDCs to hu-pDCs. Monocyte-derived human dendritic cells (hu-cDCs) and plasmacytoid dendritic cells (hu-pDCs) isolated from the same donor were infected with HSV-1 at an MOI of 5. At 6 hpi, cells were harvested, RNA isolated and subject to qRT-PCR looking at the relative expression of the HSV-1 viral gene UL54. Error bars represent the difference between triplicate assays. (c.) Dose-response HSV-1 infection of hu-pDCs. Hu-pDCs were infected with KOS and vhs- viruses at MOIs of 10 and 50; Cells were harvested at 6 hpi and subject to ELISA assay for released IL-6 and TNF-Î±. Error bars represent the difference between duplicate assays. (d.) Pro-inflammatory cytokine mRNA levels are negatively regulated by vhs in cDCs, but not in pDCs. Same as in (b.), qRT-PCR was used to measure relative gene expression of IL-6, TNF-Î±, and IL-12p40. Error bars represent the difference between triplicate assays. (1.06 MB PDF) [file pone.0008684.s004.pdf]

Supplementary Fig. 5

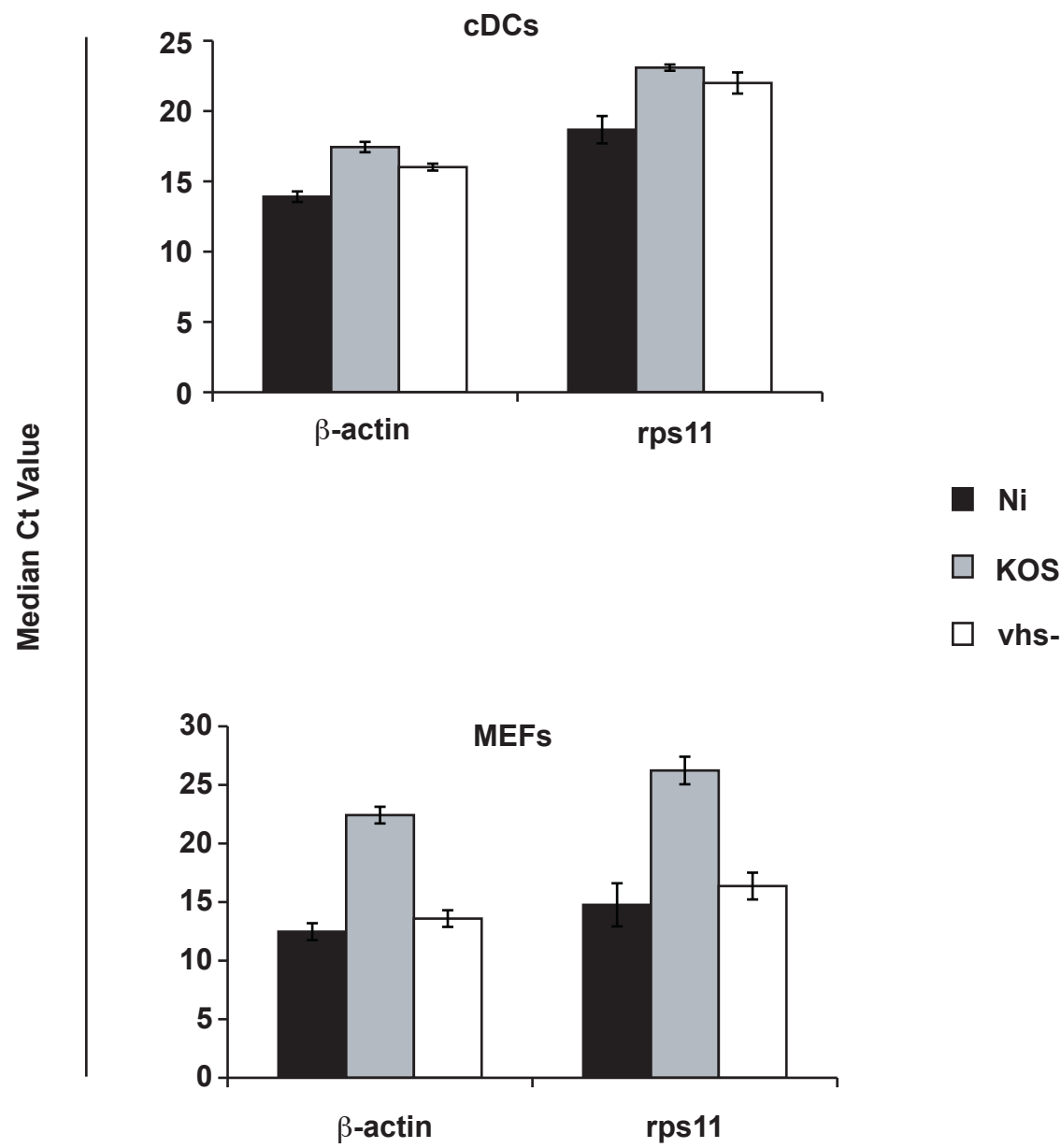

Supplement: Figure S5 — Hu-cDCs and Mouse Embryo Fibroblast (MEF) cells were infected with KOS and vhs- viruses at an MOI of 5. At 18 hpi, cells were harvested, RNA was isolated and subjected qRT-PCR. Levels of the housekeeping genes rps11 and β-actin were measured. Data shown is a representative from multiple experiments and presented as median Ct value. Error bars represent the difference between triplicate assays. (1.00 MB PDF) [file pone.0008684.s005.pdf]
